# Spontaneous regression of advanced transverse colon cancer with deficient mismatch repair: a case report

**DOI:** 10.1186/s40792-023-01595-x

**Published:** 2023-04-25

**Authors:** Shinnosuke Harata, Hiroki Takahashi, Nanako Ando, Akira Kato, Kaori Watanabe, Takeshi Yanagita, Takuya Suzuki, Hajime Ushigome, Kazuyoshi Shiga, Ryo Ogawa, Yoichi Matsuo, Akira Mitsui, Masahiro Kimura, Shuji Takiguchi

**Affiliations:** Department of Gastroenterological Surgery, Nagoya City University Graduate School of Medical Sciences, Kawasumi 1, Mizuho-cho, Mizuho-ku, Nagoya, 467-8601 Japan

**Keywords:** Spontaneous regression, Transverse colon cancer, Mismatch repair deficient (dMMR), Laparoscopic surgery

## Abstract

**Background:**

Spontaneous regression (SR) of cancer occurs in 1 in 60,000–100,000 patients. This phenomenon has been reported in almost all cancer types, most commonly neuroblastoma, renal cell carcinoma, malignant melanoma, and lymphoma/leukemia. However, SR in colorectal cancer (CRC) is extremely rare, particularly in advanced cases. Hence, this report describes a very rare case of spontaneous regression of advanced transverse colon cancer.

**Case presentation:**

A 76-year-old female with anemia was diagnosed with a type II well-differentiated adenocarcinoma in the middle transverse colon. Two months later, a second colonoscopy examination was performed for preoperative marking, and it revealed tumor shrinkage and a shift to type 0–IIc morphology. Endoscopic tattooing was then performed, followed by a laparoscopic partial resection of the transverse colon with D3 lymph node dissection. However, the resected specimen contained no tumor, and colonoscopy showed no tumor remnants in the remaining colon. Histopathological examination revealed mucosal regeneration and a mucus nodule in between the submucosal and muscular layers, with no cancer cells detected. Immunohistochemical analysis revealed the loss of MutL homolog 1 (MLH1) and postmeiotic segregation increased 2 (PMS2) expression in the cancer cells of biopsied specimens, suggesting deficient mismatch repair (dMMR). The patient continues to be followed up until 6 years postoperatively, and no recurrence has been observed. In this study, we also reviewed similar reported cases of spontaneous regression of cancer involving dMMR.

**Conclusion:**

This study presents a rare case of spontaneous regression of advanced transverse colon cancer wherein dMMR is strongly involved. However, further accumulation of similar cases is needed to elucidate this phenomenon and to develop new treatment strategies for CRC.

## Background

Spontaneous regression (SR) of malignant tumors reportedly occurs in 1 per 60,000–100,000 patients with cancer [1]. SR has been reported in virtually all types of human cancer, most especially neuroblastoma [2], renal cell carcinoma [3], malignant melanoma [4], and lymphoma/leukemia [5, 6]. However, SR of colorectal cancer (CRC) is extremely rare, particularly in advanced cases. According to our literature search, SR of advanced CRC has been reported in only seven cases in the English literature since 2000 [7–13]. The exact cause of this SR is unknown. However, very recently, the association of SR with deficient mismatch repair (dMMR) has gradually become apparent [12, 13]. Here, we report a very rare case of SR of advanced transverse colon cancer with dMMR involvement. We also reviewed all the seven similar reported cases.

## Case presentation

A 76-year-old female was admitted to our hospital with anemia. Her previous medical history included endoscopic submucosal dissection 1 year prior for early gastric cancer, appendectomy, and hypertension. The patient was postmenopausal, with no family history of cancer.

**Fig. 1:**
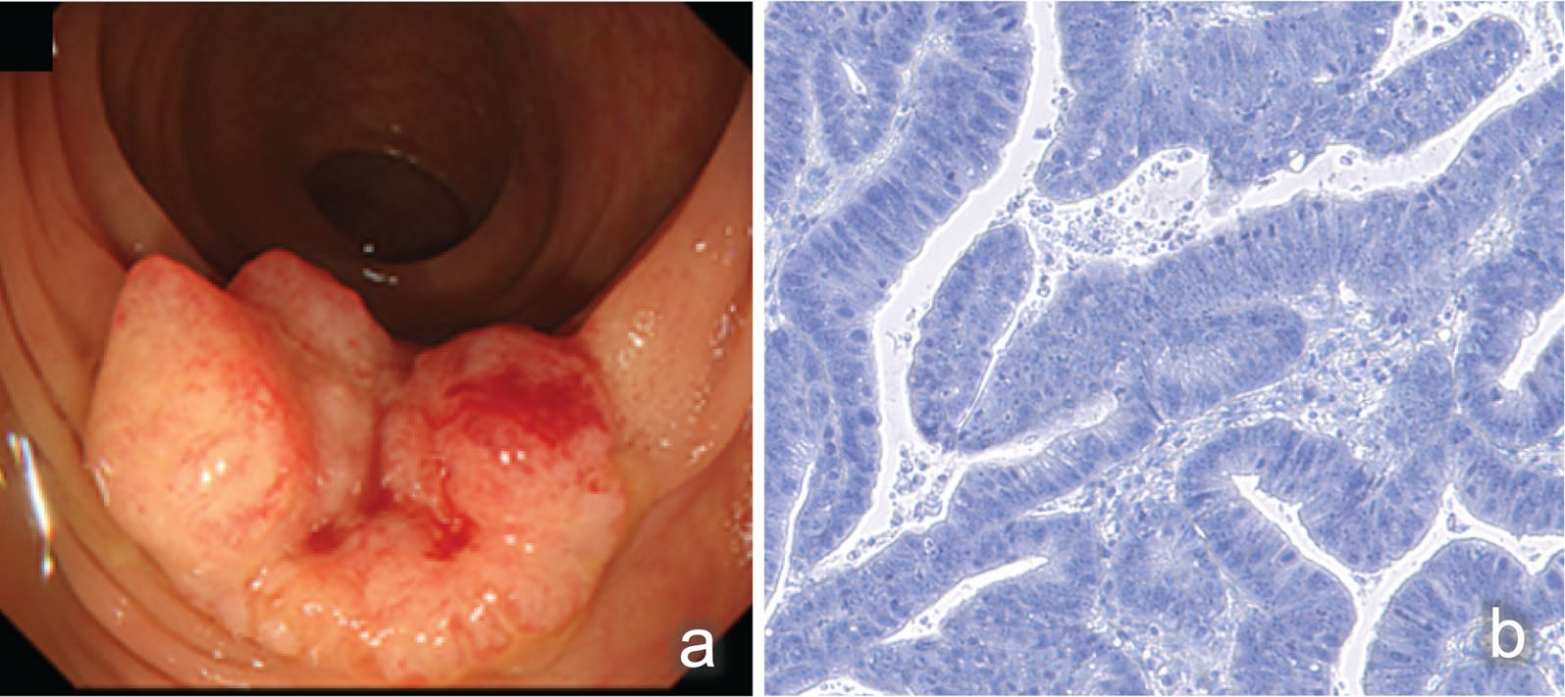
Initial examination findings of colonoscopy. **a** Type II tumor, measured 30 mm in diameter and located in the transverse colon. **b** The biopsy specimen demonstrated a well-differentiated adenocarcinoma

**Fig. 2:**
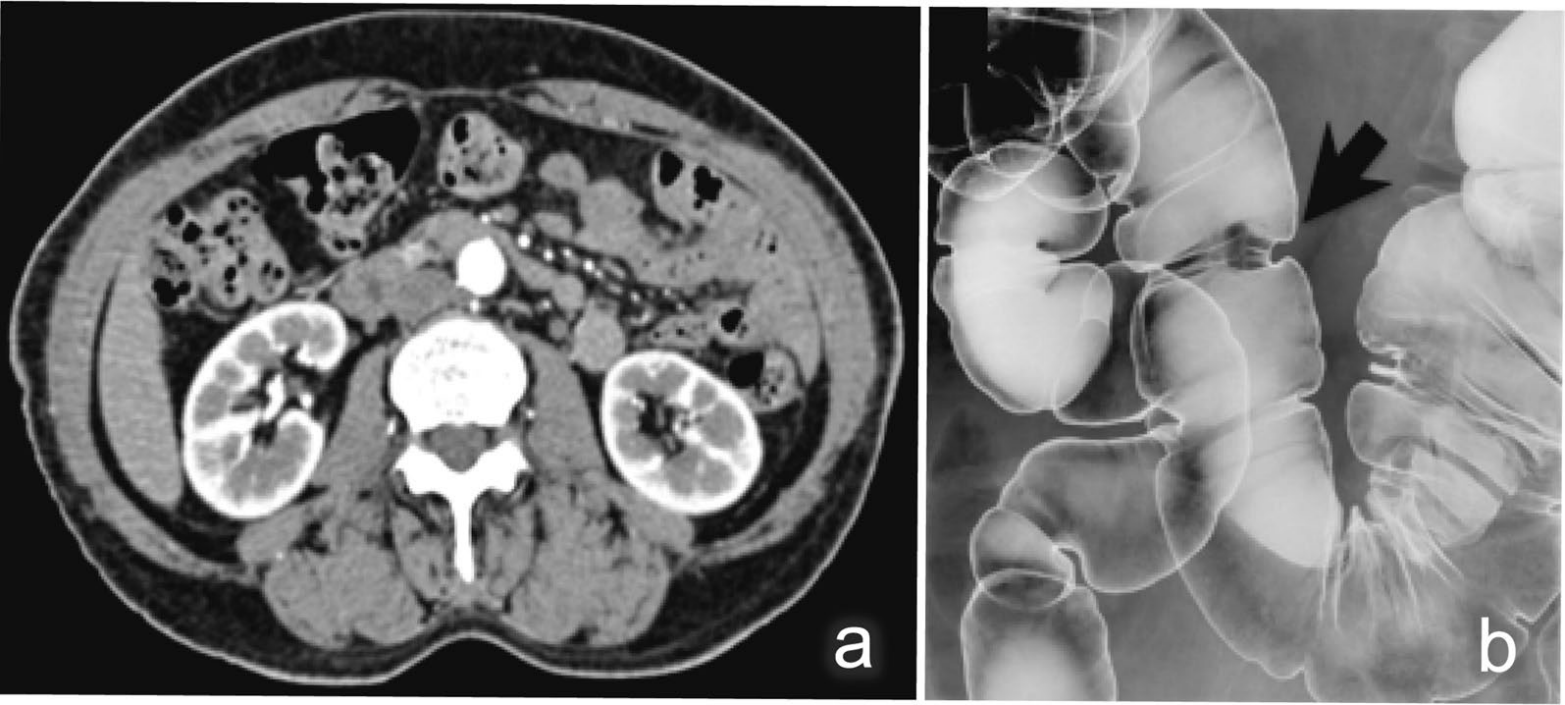
Individual imaging findings during the initial examination. **a** Computed tomography revealed no remarkable abnormality and no evidence of wall thickening in the transverse colon, lymph node metastasis, and distant metastasis. **b** Barium enema examination revealed an arc-shaped deformation in the transverse colon, confirming that the lesion was a type II tumor

**Fig. 3:**
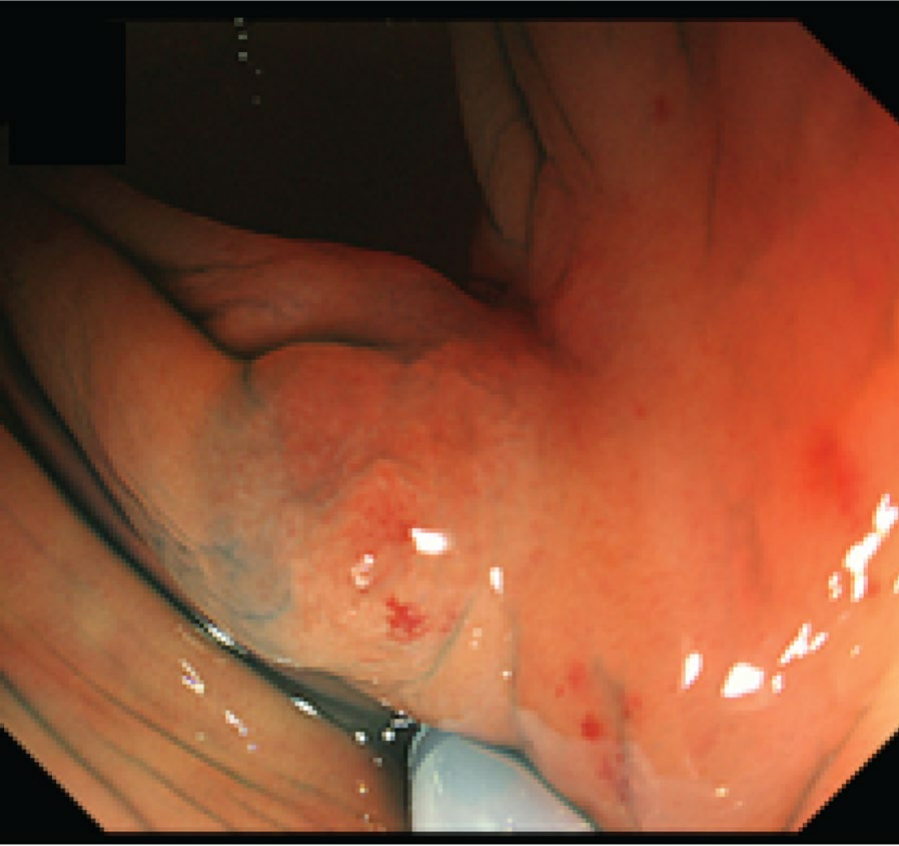
Second examination findings of colonoscopy. Colonoscopy was performed 2 months after the initial one for preoperative localization, and revealed tumor shrinkage to a diameter of 15 mm and a shift to type 0–IIc morphology

**Fig. 4:**
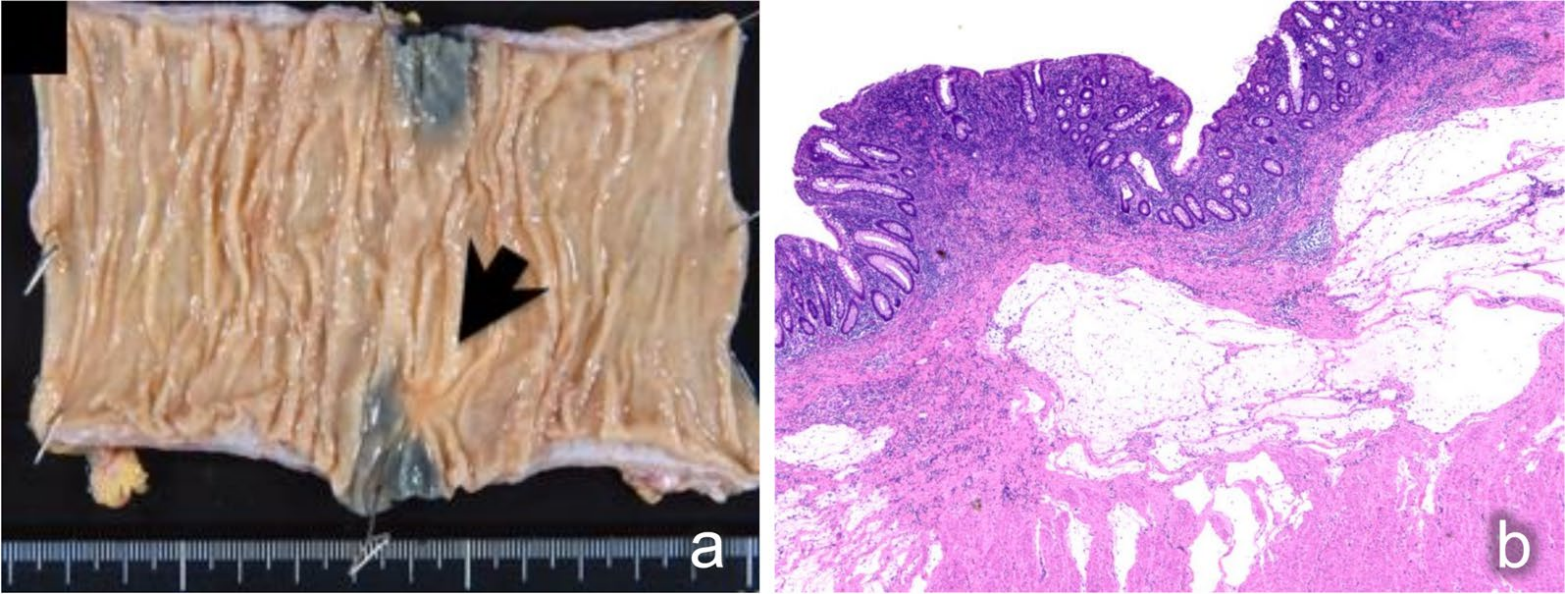
Resected specimen and histopathological examination findings. **a** The resected specimen left only a discolored scar, and the tumor had disappeared. **b** Histopathological examination revealed mucosal regeneration and a mucus nodule in between the submucosal and muscular layers, with no cancer cells. All of the resected specimens also showed no malignancy

**Fig. 5:**
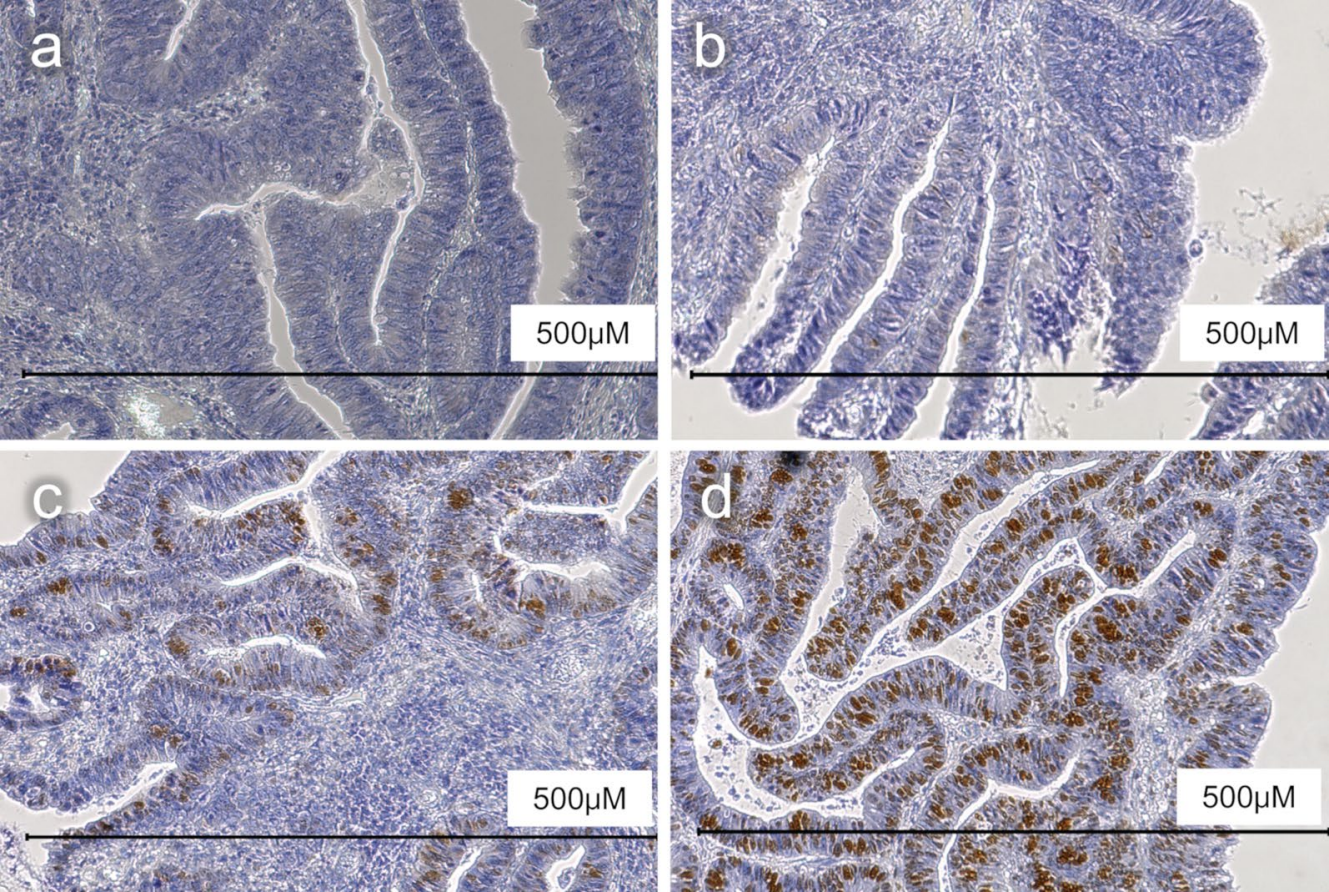
Immunohistochemical analysis of MMRs. PMS2 (**a**), MLH1 (**b**), MSH2 (**c**), and MSH6 (**d**)

Laboratory examinations revealed pancytopenia (white blood cell count, 2200/μL; hemoglobin count, 9.2 g/dL; and platelet count, 75,000/μL), suggesting myelodysplastic syndrome. The patient was not treated for pancytopenia, and the treatment plan was observation. The carcinoembryonic antigen and carbohydrate antigen 19-9 (CA19-9) levels were 9.3 ng/mL (normal: < 5.0 ng/mL) and 6.2 U/mL (normal: < 37 U/mL), respectively. Colonoscopy revealed a type II tumor, which measured 30 mm in diameter and was located in the middle transverse colon (Fig. 1a). Additionally, biopsy demonstrated a well-differentiated adenocarcinoma (Fig. 1b). However, computed tomography detected no remarkable abnormality and no evidence of lymph node metastasis and distant metastasis (Fig. 2a). Moreover, barium enema examination revealed an arc-shaped deformation in the transverse colon, further indicating that the lesion was a type II tumor (Fig. 2b). According to the TNM classification, the clinical diagnosis was cT2N0M0 (Stage I) [14]. Two months after the initial colonoscopy, the patient underwent colonoscopy again for preoperative localization, revealing tumor shrinkage to a diameter of 15 mm and a shift to type 0–IIc morphology (Fig. 3). After confirming that the tumor was in the correct location, we performed endoscopic tattooing, followed by a laparoscopic partial resection of the transverse colon with D3 lymph node dissection. The resected specimen left only a discolored scar, and the tumor had disappeared (Fig. 4a). Histopathological examination revealed mucosal regeneration and a mucus nodule in between the submucosal and muscular layers, with no any residual cancer cells noted. All of the resected specimens also demonstrated no malignant lesions (Fig. 4b). Similarly, the dissected lymph nodes showed no cancer cells. Considering that the relationship between SR and dMMR has been pointed out, we further performed immunohistochemical analysis using the biopsy specimens. Results showed that MutL homolog 1 (MLH1) and postmeiotic segregation increased 2 (PMS2) were not expressed, suggesting dMMR (Fig. 5). Germline mutation testing was not performed because the patient did not request it. I have not measured methylation either.

Subsequently, the patient had an uneventful recovery and was discharged 7 days postoperatively. Colonoscopy was then performed 2 months postoperatively, and it showed no tumors in the colon and rectum. The patient continues to be followed up until 6 years postoperatively without any adjuvant therapy. No recurrence has been observed. Accordingly, the final diagnosis was SR of advanced transverse colon cancer.

## Discussion

As mentioned, SR is reported in virtually all types of human cancer, with neuroblastoma, renal cell carcinoma, malignant melanoma, and lymphoma/leukemia as the most common. However, SR of CRC is extremely rare, and only seven cases of SR of primary advanced CRC have been reported since 2000 (Table 1).

**Table 1:** Reported cases of SR of primary advanced CRC since 2000

No.	Author	Year	Age	Sex	Primary site	Morphological type	Size (mm)	Histology	MMR states	Duration (month)	Follow-up (year)	References
1	Sakamoto	2009	80	M	Rectum	1	25	Well	ND	3	2	[7]
2	Shimizu	2010	80	M	Transverse	1	25	Mod	ND	7	5	[8]
3	Sekiguchi	2013	69	F	Ascending	2	20	Mod	ND	1.5	5	[9]
4	Kihara	2015	64	M	Transverse	2	30	Mod	ND	1.5	1	[10]
5	Chida	2017	80	M	Transverse	2	30	Poor	ND	1	1	[11]
6	Karakuchi	2019	70	M	Transverse	2	30	Poor	dMMR	2	1	[12]
7	Nishiura	2020	67	F	Transverse	2	13	Poor	dMMR	3	5	[13]
8	Yokota	2020	64	M	Transverse	2	20	Mod	dMMR	1.5	6	[14]
9	Present case	2022	76	F	Transverse	2	30	Well	dMMR	2	6	–

*Well* well-differentiated adenocarcinoma; *Mod* moderately differentiated adenocarcinoma; *Poor* poorly differentiated adenocarcinoma; *dMMR* deficient mismatch repair; *ND* not described

These reported cases consist of five males and two females, and the median age was 73 (64–80) years. The primary tumor site was mostly on the right side of the colon, and only one case was on the left side. The tumor morphology was mostly type II, and the median tumor size was 27.5 mm. The histological findings varied, and no consistent trend was observed. In addition, the median duration of tumor resolution was 2 months.

One of the reasons for the rarity of SR of CRC is that CRC is usually treated immediately after the diagnosis [5]. Treatment is rarely provided more than a few months after CRC diagnosis. In this study, the patient was treated 2 months after being diagnosed with CRC because some examinations for pancytopenia still had to be performed.

The reported risk factors for SR include sepsis with prolonged fever evolution, invasive treatment (e.g., colostomy), psychological factors, genetic factors, and physical factors [15]. These factors might stimulate several immune reactions or endocrine systems, resulting in SR. Among these factors, physical factors, such as ischemia caused by tumor progression, mechanical stimulation of intestinal peristalsis, circulatory failure caused by distortion or tow by tumor, and tissue biopsy, are involved in the SR of alimentary tract cancer [8]. In this study, preoperatively, the patient did not undergo any treatment except for the tissue biopsy; therefore, tissue biopsy is the only possible factor causing SR of CRC.

Very recently, the relationship between the SR of CRC and dMMR has been pointed out [12, 13]. Mismatch repair is a process involving many proteins in very highly conserved cells to identify and repair mismatched bases. In fact, dMMR, which is found in 15% of patients with CRC (12% sporadic cases and 3% hereditary cases) [16], results in a strong mutant phenotype called microsatellite instability (MSI), which is characterized by an extensively long polymorphism of microsatellite sequences caused by DNA polymerase slippage [17]. By using immunohistochemistry to test the MMR proteins such as MLH1, MutS homolog 2 (MSH2), MSH6, and PMS2, we can indicate the presence or absence of a functional MMR system and thus indirectly, MSI [18].

Microsatellite instability-high (MSI-H)/dMMR tumors correlate with an increase in neoantigens, which are newly formed antigens derived from genetic mutations in cancer cells. In stages II and III CRC, especially stage II, dMMR CRC is a favorable factor for recurrence and prognosis [19]. Furthermore, dMMR CRC has several characteristics, including older age, female sex, right-sided colon, and a high incidence of poorly differentiated adenocarcinoma or mucinous adenocarcinoma. A review of eight cases showed that SR of CRC was consistent with dMMR CRC characteristics, including older age, right-sided colon, and a high incidence of poorly differentiated adenocarcinoma. Finally, we speculated that SR of CRC was caused by a strong immune response induced by stimuli such as tissue biopsy against the background of dMMR. Some dMMR-related tumors are associated with Lynch syndrome, and patients with Lynch syndrome have been reported to show increased mucosal T-cell infiltration even in the absence of cancer [20]. The patient did not wish to be examined and was not examined closely for Lynch syndrome.

When encountering SR of CRC, we need to pay attention to the following points. First, we need to double-check that we are not misidentifying patients. In this case, we confirmed that we never had patient misidentification; we even revalidated our endoscopic system. Next, resection is still required, even if the tumor has disappeared preoperatively. The surgical resection was appropriate because we found no evidence of an absence of cancer cells in the deeper layer compared with that in the submucosal layer. Moreover, tumor regrowth after its remission may be caused by spontaneous decapitation [9]. Nishiura et al. reported a case of SR of advanced transverse colon cancer with remaining lymph node metastasis [13]. Finally, regular follow-up is crucial in SR cases. For these reasons, regular follow-up is necessary, even if SR of CRC occurs. Our patient continues to be followed up until 6 years postoperatively, with no observed recurrence.

## Conclusions

This report describes a rare case of SR of advanced transverse colon cancer, and dMMR is strongly involved. However, further accumulation of similar cases is necessary to elucidate this phenomenon to develop new treatment strategies for CRC.

## Data Availability

The data generated or analyzed in this study are included in a published article.

## Abbreviations

CRC: Colorectal cancer
dMMR: Deficient mismatch repair
SR: Spontaneous regression
MLH1: MutL homolog 1
MSH2: MutS homolog 2
MSI-H: Microsatellite instability-high
PMS: Postmeiotic segregation increased 2
